# Comparison of Efficacy of Deep Brain Stimulation of Different Targets in Parkinson's Disease: A Network Meta-Analysis

**DOI:** 10.3389/fnagi.2019.00023

**Published:** 2019-02-22

**Authors:** Zhiqi Mao, Zhipei Ling, Longsheng Pan, Xin Xu, Zhiqiang Cui, Shuli Liang, Xinguang Yu

**Affiliations:** Department of Neurosurgery, Chinese PLA General Hospital, Beijing, China

**Keywords:** Parkinson's disease, deep brain stimulation, subthalamic nucleus, internal globus pallidus, pedunculopontine nucleus, ventral intermediate nucleus, network meta-analysis

## Abstract

**Background:** Deep brain stimulation (DBS) is considered an effective treatment option for Parkinson's disease (PD). Several studies have demonstrated the efficacy of neurostimulation in patients with advanced PD. The subthalamic nucleus (STN), the internal globus pallidus (GPi), ventral intermediate nucleus (Vim), and pedunculopontine nucleus (PPN) are reportedly effective DBS targets for control of Parkinsonian tremors. However, there is no consensus on the ideal target for DBS in patients with Parkinson's disease. Only a few studies have directly compared the efficacy of DBS of the Vim, STN, and GPi. Therefore, we searched PubMed, Embase, Cochrane Library, and other databases for observational studies, extracted data on unified Parkinson's disease rating scale (UPDRS) scores and performed a comprehensive network meta-analysis of different strategies of DBS and compared the efficiency of DBS at different targets.

**Methods:** Forest plot was used to examine the overall efficiency of DBS; cumulative probability value was used to rank the strategies under examination. A node-splitting model was employed to assess consistency of reported outcomes inconsistency. A total of 16 studies which focused on UPDRS improvement were included in the network meta-analysis.

**Results:** By comparing the overall efficiency associated with each target, we confirmed the efficacy of DBS therapy in PD. Our findings revealed similar efficacy of DBS targeted at GPi and STN in the on-medication phase [GPi-3.9 (95% CI −7.0 to −0.96); STN-3.1 (−5.9 to −0.38)]; however, in the off-medication phase, Vim-targeted DBS was associated with better improvement in UPDRS scores and could be a choice as a DBS target for tremor-dominant Parkinsonism.

**Conclusions:** Our findings will help improve clinical application of DBS.

## Introduction

Parkinson disease (PD) is a chronic, progressive, debilitating neurodegenerative disease which affects an estimated 1% of the population aged above 55 years. The condition has a substantial impact on the physical, psychological, and social health of the afflicted individual (Ascherio and Schwarzschild, 2016). PD is characterized by dysfunction of motor system, including tremors, muscle stiffness, restricted mobility, and difficulty with balance (Samii et al., 2004; Rodriguez-Oroz et al., 2009). PD is one of the most challenging chronic geriatric disorders, which affects a patient's quality of life and imposes a substantial burden on caregivers.

Levodopa is the principal pharmaceutical treatment for PD at early stage. However, efficacy of pharmacological treatment in alleviating motor dysfunction tends to decrease over time, with a concomitant increase in incidence of side effects, such as dyskinesias and psychotic symptoms. For instance, patients with advanced PD often exhibit significantly rapid and unpredictable swings between mobility (on-medication phase) and immobility (off-medication phase), frequently along with L-dopa-induced dyskinesia, and immobility (the off phase) (Maetzler et al., 2009). Many such patients suffer from unsatisfactory to adjusted pharmacological therapy, which progressively compromises their quality of life. Therefore, many non-pharmacological methods, such as deep brain stimulation (DBS), have been developed and studied to overcome this challenge.

Deep brain stimulation (DBS) is a well-established surgical treatment modality for PD. DBS has been used to treat neuropsychiatric and neurologic symptoms in patients who do not respond well to medical therapy. However, the mechanism of action of DBS is not clear (Benabid, 2003). The treatment involves electrical stimulation of specific parts of the brain with the objective to block the aberrant nerve signals responsible for the symptoms (Fasano et al., 2012). With stereotactic implantation of an electrode in a pre-selected brain target site, the electrode parameters can be controlled using a neuromodulator implanted under the patient's skin. DBS therapy has been shown to be superior to the best medical therapy in improving motor function and quality of life of patients who do not adequately respond to drugs, or who have motor complications (Rosin et al., 2011).

The most commonly used targets of DBS are the internal globus pallidus (GPi) and subthalamic nucleus (STN), even though pedunculopontine nucleus (PPN) and posterior subthalamic area (PSA) are reportedly effective targets for parkinsonian tremor control (DeLong and Wichmann, 2015; Katz et al., 2015). However, there is no universal consensus on the best target for DBS in patients with Parkinson's disease. Prospective randomized trials have shown comparable clinical improvement with STN DBS and GPi DBS (Rothlind et al., 2015). In the last two decades, several studies have sought to compare treatment outcomes associated with different therapeutic targets (Lukins et al., 2014). Among these studies, almost all of the treatments were examined at the same level of objective evidence (Schuurman et al., 2008; St George et al., 2012). However, very few studies have directly compared the efficacy of DBS of the ventral intermedius nucleus (Vim), STN, and GPi. Therefore, a comprehensive review of different strategies for DBS and a comparison of their efficiency is of much clinical relevance. In this work, we highlight the important aspects of this therapy and present the findings of a network meta-analysis to determine the best therapeutic targets for patients with PD. The objective of this research is to provide new insights that extend beyond the specific therapeutic strategy itself.

## Materials and Methods

### Literature Search

Electronic bibliographic databases were searched for relevant clinical trials: English databases included Pubmed, EMBASE, The Cochrane Library (Cochrane Database of Systematic Reviews), Cochrane Central Register of Controlled Trials (CENTRAL), Health Technology Assessment Database, and Web of Science (science and social science citation index); the Chinese databases included Technology of Chongqing VIP database, SinoMed, Wan Fang Data, and China National Knowledge Infrastructure (CNKI).

The search terms were adapted for use with other bibliographic databases in combination with database-specific filters for controlled trials, wherever these were available. The publication date was from January 1990 to June 2015. The search terms used were “Parkinson's disease/PD,” “Deep brain stimulation/DBS,” “treatment,” and “unified Parkinson's disease rating scale (UPDRS).”

### Inclusion and Exclusion Criteria

For our analysis, we included observational clinical studies that compared GPi-DBS vs. STN-DBS, or GPi vs. medical therapy, or STN-DBS vs. medical therapy in patients with advanced PD.

The following inclusion criteria were used: (1) clinical trials of DBS for treatment of idiopathic PD; (2) study subjects: patients clinically confirmed as PD; (3) study outcomes: studies that used the UPDRS score to assess the post-treatment results; (4) outcomes in those studies were measured more than 3 months post-surgery and contained clear reports of medication phases.

The exclusion criteria were: (1) DBS was performed in patients with pathologies other than PD; (2) they were not concurrent, controlled clinical studies; (3) data could not be extracted or lacked data integrity; (4) involved complex intervention strategies.

### Quality Assessment and Data Extraction

The Newcastle-Ottawa Quality Assessment Scale was used to assess the methodological quality of the prospective or retrospective cohort studies. Three major components of each study were examined: patient selection; comparability of the intervention and the observation groups; and outcome assessment. Controversial items were discussed with the primary investigator before final consensus was reached. The final selected studies were independently assessed by two reviewers using a standardized form. Data on following variables were retrieved from these articles: name of first author, publication year, sample size, UPDRS scores, and DBS targets.

### Efficacy Measures

The UPDRS is widely used for longitudinal assessment of functional status and motor performance of patients with PD. The UPDRS comprises of following sections: UPDRS I measures mentation, behavior, and mood; UPDRS II is used for self-evaluation of the activities of daily life (ADLs), including, speech, swallowing, handwriting, dressing, hygiene, falling, salivating, turning in bed, walking, and cutting food; UPDRS III measures clinician-scored motor function; and UPDRS IV measures complications from therapy. Higher UPDRS scores correlated with more severe PD. UPDRS III motor score was the primary score measured in studies on treatment of PD. Therefore, the primary therapeutic efficiency of the included studies were estimated using the UPDRS III scores (0–108) to assess its improvement in PD symptoms.

### Statistical Analysis

The conventional pair-wise meta-analysis and network meta-analysis were carried out according to the Bayesian framework by using the R3.2.3 software. UPDRS score was utilized to compare the efficacy of various treatments; mean difference and 95% credible intervals (CI) with a significance level at 0.05 are reported. Both direct and indirect comparisons were included and the results illustrated as forest plots. Additionally, the surface under cumulative ranking curve (SUCRA) was adopted to assess rank probabilities with respect to each clinical outcome. A higher rank probability value indicates a more desirable property with respect to a certain endpoint. Heterogeneity among the included studies was assessed according to Cochran's Chi-squared statistic and *I*^2^ test. Deek's funnel plots were adopted to investigate publication bias.

## Results

### Study Characteristics

A total of 206 studies were retrieved on initial literature search. As shown in Figure S1, 224 records were retrieved from the database by searching relevant keywords; 15 duplicates were removed, and 151 literatures were excluded as they did not qualify the inclusion criteria. According to exclusion criteria, only 58 out of the remaining 209 studies were finally included in this network meta-analysis. After exclusion of irrelevant literature including reviews, case reports, comments, editorials, animal studies, and basic trials, 16 studies met our criteria. Finally, our meta-analysis comparing the efficiency of STN, GPi, PPN, Vim DBS, and medical therapy (MT) included 16 studies with a combined study population of 1,252 patients with PD (Table 1, Figure S2).

**Table 1:** Characteristics of studies included in the analysis.

**References**	**Country**	**PD duration (y)**		**Intervention**		**Sample Size**		**Mean Age (y)**	
		**1**	**2**	**1**	**2**	**1**	**2**	**1**	**2**
Anderson et al., 2005	USA	10.3	15.6	GPi	STN	11	12	54	61
Katayama et al., 2000	Japan	NA	NA	GPi	STN	7	11	NA	NA
Deep-Brain Stimulation for Parkinson's Disease Study Group et al., 2001	USA	13.5	14	GPi	STN	36	91	59	55.7
Peppe et al., 2010	Italy	16	13.6	PPN	STN	5	5	57.8	62
Follett et al., 2010	USA	11.5	11.1	GPi	STN	152	147	61.8	61.9
Zahodne et al., 2009	USA	12.5	13.5	GPi	STN	22	20	61.3	61.3
Khan et al., 2011	UK	21.0	19.1	PPN	Vim	7	7	60.7	60.7
Parihar et al., 2015	USA	10.5	9.8	Vim	STN	8	10	54.7	53.6
St George et al., 2015	Australia	15.4	12.1	GPi	MT	10	8	62.8	60
Odekerken et al., 2013	Netherlands	8.0	9.0	GPi	STN	65	63	52.9	60.3
Okun et al., 2012	USA	12.1	11.7	STN	MT	101	35	60.6	59.5
Robertson et al., 2011	USA	15.1	16.8	GPi	STN	13	14	65.5	63.8
Rocchi et al., 2012	USA	12.9	11.9	GPi	STN	14	15	61.1	61.4
Rothlind et al., 2015	USA	11	12.8	GPi	MT	80	116	61.3	62.3
Parihar et al., 2015	USA	10.5	9.8	Vim	STN	8	10	54.7	53.6
Weaver et al., 2012	USA	11.4	11.3	GPi	STN	89	70	60.4	60.7

*STN, subthalamic nucleus; GPi, internal globus pallidus; Vim, ventral intermediate nucleus; PPN, pedunculopontine nucleus (PPN)*.

### Changes in UPDRS III Scores and Traditional Meta-Analysis

**Figure 1:**
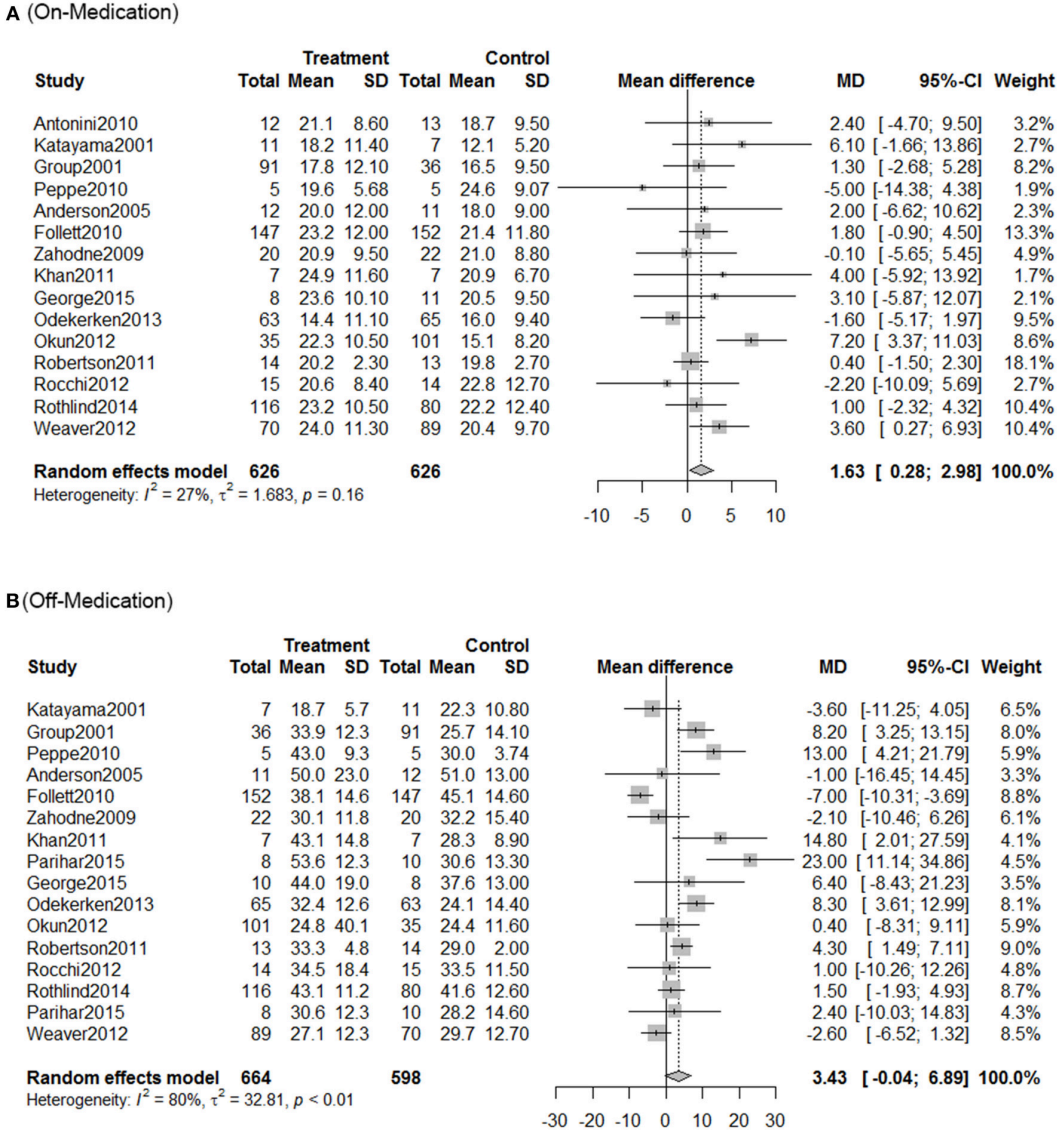
Meta-analysis for DBS in PD treatment. Forest plots of standardized mean difference in DBS treatment and control during on-medication period **(A)** and off-medication period **(B)**. SD, standardized mean; CI, confidence interval; MD, mean difference.

Using traditional pair-wise meta-analysis to **c**ompare the overall efficiency of DBS therapy, a significant benefit in favor of DBS was observed in both on- and off-medication phases. These results showed that the mean reduction in UPDRS motor scores was comparable with that achieved with medical therapy. During the on-medication phase, DBS treatment significantly reduced UPDRS III scores; the overall pooled standardized mean differences (SMD) was 1.63 (95% CI: 0.28–2.98). In the off-medication phase, the overall pooled SMD outcome value was 3.43 (95% CI: 0.04–6.89, *p* < 0.01). On assessment of heterogeneity using the Chi-squared and *I*-square tests, mild heterogeneity was observed among the treatment groups [τ2 = 32.8; *I*^2^ = 80%]. There was no significant heterogeneity among the included studies (Figure 1).

### Network Meta-Analysis

**Figure 2:**
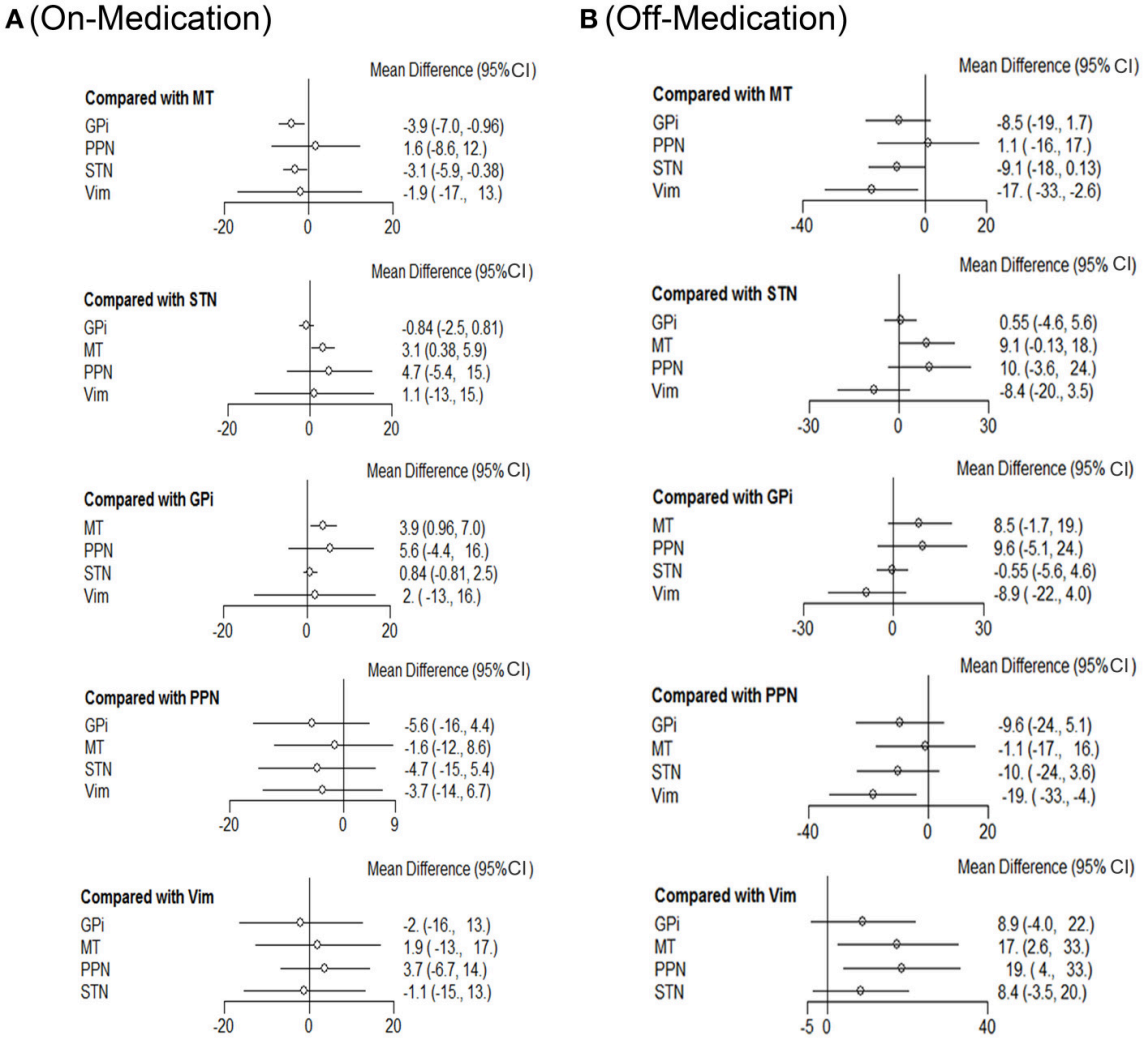
Comparison of efficiency on different DBS targets. Network meta-analysis plot for primary outcomes of different DBS targets during on-medication period **(A)** and off-medication period **(B)**. STN, subthalamic nucleus; GPi, internal globus pallidus; Vim, ventral intermediate nucleus; PPN, pedunculopontine nucleus; MT, medical therapy.

Next we performed network meta-analysis to explore if there were differences with different targets of DBS therapy (Figure 2). In the on-medication phase, with efficacy of medical therapy (MT) as the standard comparison, the mean differences were: GPi-3.9 (95% CI −7.0 to −0.96); PPN 1.6 (−8.6 to 12); STN-3.1 (−5.9 to −0.38); Vim-1.9 (−17 to 13).

In off-medication phase, the mean differences were: GPi-8.5 (95% CI −19 to 1.7); PPN 1.1 (−16 to 17); STN-9.1 (−18 to −0.13); Vim-17 (−33 to −2.6).

STN and GPi appear to be more effective than the other DBS targets with respect to efficiency. Besides, Vim also showed promising results concerning this clinical outcome during the off-medication phase and would be a promising candidate in DBS therapies for patients with no pharmacological treatment.

### Rank Probability

**Figure 3:**
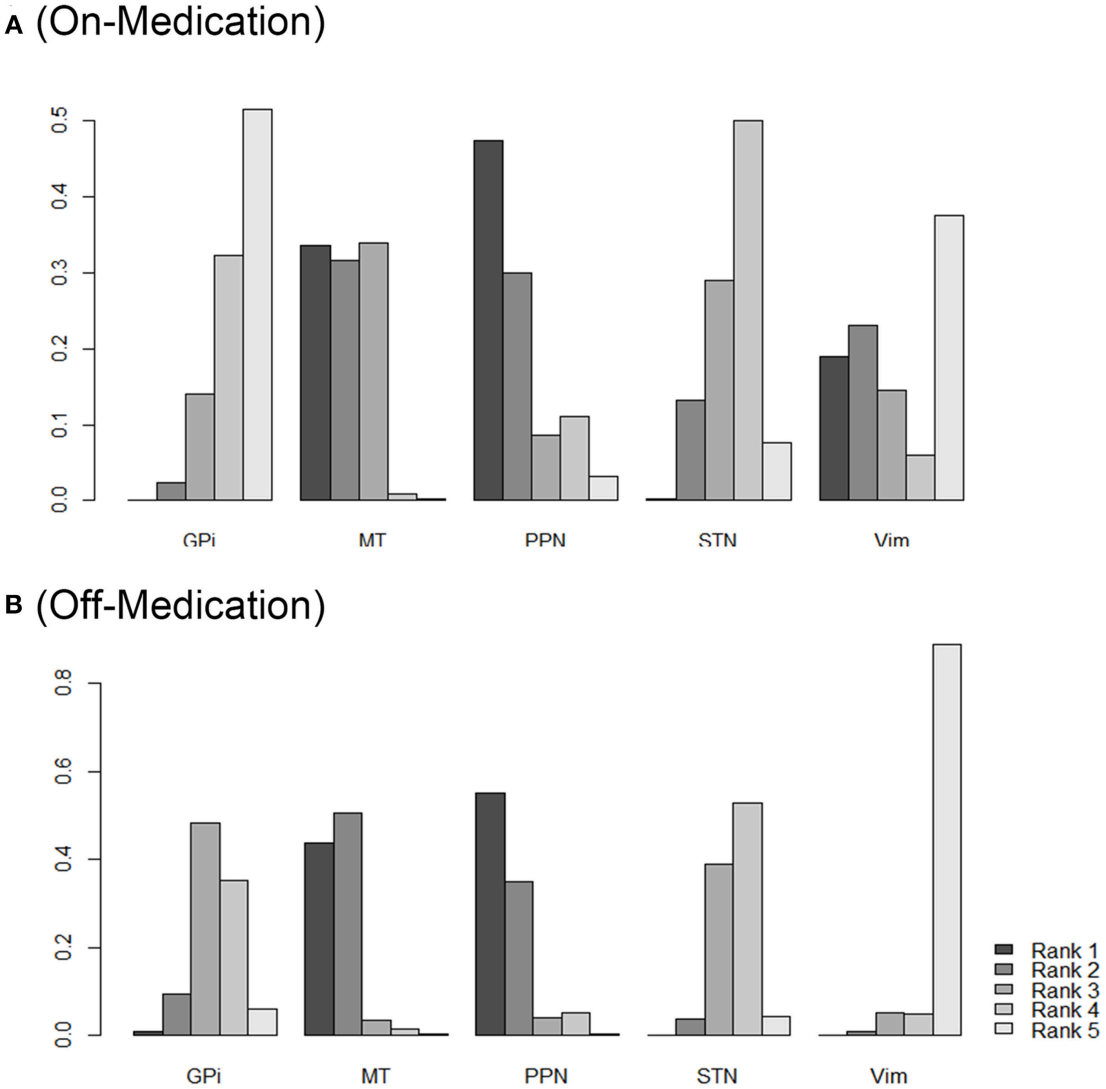
Rank probability of different DBS targets as measured by the outcomes during on-medication period **(A)** and off-medication period **(B)**. Network ranking was used to measure the probability of the best treatment among different DBS targets, the figure shows the most likely ranking from rank5 (best treatment) to rank1 (least effective treatment), higher rank probability indicates better outcomes. STN, subthalamic nucleus; GPi, internal globus pallidus; Vim, ventral intermediate nucleus; PPN, pedunculopontine nucleus; MT, medical therapy.

As shown in Figure 3, the SUCRA values and rank probability of the efficacy against different targets of DBS demonstrated that GPi ranked the highest (85.7%) in the on-medication phase, while Vim ranked the highest during the off-medication phase (76.4%).

Figure 3 provides the ranking plot showing probability of each target strategy ranked in terms of efficiency; furthermore, rank probability results provide for further comparison among different DBS targets. In general, GPi and STN showed a more prominent efficiency compared to that at other sites; PPN, in contrast, showed the least effectiveness in both the on-medication and the off-medication phases.

### Consistency and Convergence Analysis

To assess for inconsistency among the included studies, node-splitting models were used for testing the difference between the direct and indirect comparisons (Figure S3). The goal was to determine the agreement between the direct and indirect evidence on a specific node (the split node). After constructing the node-splitting models, no significant inconsistency was observed. The results of the consistency model were reliable. Moreover, PSRF values of all parameters were limited to 1, which demonstrated good convergence and efficiency. The comparison-adjusted funnel plot of the efficacy of these 16 treatments showed that there was no publication bias among the included studies.

## Discussion

DBS treatments for advanced PD have shown significant improvement in non-motor and motor functions. To identify the best target of DBS, several studies have compared the efficiency of different DBS targets (such as the STN and GPi) which have shown apparent benefit in reducing motor syndromes (Schuepbach et al., 2013). Moreover other more novel targets such as PPN are currently being explored (Welter et al., 2015). Reports of differences in outcomes associated with different targets are very limited. Further, most studies have compared the difference in outcomes only between the GPi and STN targets.

To assess the best DBS target for treatment of PD, we performed a network meta-analysis based on data from16 cohort studies (combined *n* = 1,252), most of which compared different DBS targets or DBS vs. MT. Motor control is the main therapeutic goal of advanced PD patients who receive DBS treatment. Therefore, in this study we analyzed the UPDRS-III scores at 3 months to 3 years follow-up points post-surgery. The main finding of our study is that among the different DBS targets, GPi and STN showed better efficacy than other targets. Furthermore, the studies also confirmed that if DBS was used alone, its efficiency was not as good as that of the standard medical therapy. Therefore, it is better to combine DBS with medical therapy other anti-Parkinson drugs.

We observed no difference between STN and GPi DBS with respect to improvement, which is consistent with the results of a previous study (Liu et al., 2014). However, Vim-targeted DBS was associated with better improvement in UPDRS scores in the off-medication phase, which suggests that Vim DBS is superior in terms of allowing greater reduction in medication after DBS and that Vim could be a candidate target for DBS in patients with tremor-dominant Parkinsonism. However, these results should be interpreted with caution as the small sample size in these studies may have introduced an element of bias.

Moreover, our study has some other potential weaknesses. Firstly, significant heterogeneity was observed in the included studies. This is likely attributable to differences between studies with respect to measurements, DBS techniques, and postoperative management of patients. Secondly, 3 of the included studies had smaller sample sizes of patients (*n* < 10) as compared to the other studies, which may also have introduced an element of bias. Thirdly, more primary outcomes should be examined in our analysis, such as quality of life and emotional conditions. Finally, only studies published in English were included in this analysis, which is another potential source of bias. Considering the limitations described above, due caution should be exercised while interpreting our findings.

DBS has been shown to be an effective therapy for specific patients with advanced PD. There have been rapid developments and progress in neuroengineering, and new DBS stimulation delivery systems are being developed to improve the efficacy and effectiveness of this therapy (Martinez-Ramirez et al., 2015). In fact, there is no one approach that is suitable for all patients. Clinicians should match the individual patient's symptoms with appropriate DBS target for amelioration of specific symptoms.

## Author Contributions

ZM and XY: designed the study; ZM, LP, XX, ZC, and SL: contributed to the literature research, the study selection, and the data extraction; ZL: checked the data and information checking; XY: performed the quality assessment; LP, ZM, XX, and SL: analyzed the data. All authors reviewed the manuscript.

### Conflict of Interest Statement

The authors declare that the research was conducted in the absence of any commercial or financial relationships that could be construed as a potential conflict of interest.

## Supplementary Material

Figure S1 Schematic illustration of the literature search and study selection criteria.

Figure S2 Evidence network for different DBS (Deep brain stimulation) targets. STN, subthalamic nucleus; GPi, internal globus pallidus; Vim, ventral intermediate nucleus; PPN, pedunculopontine nucleus; MT, medical therapy.

Figure S3 STN-subthalamic nucleus, GPi-internal globus pallidus, Vim-ventral intermediate nucleus, PPN-pedunculopontine nucleus, MT-medical therapy, sd-standardized mean.
