# Supplementary figures and images for: Comparison of Efficacy of Deep Brain Stimulation of Different Targets in Parkinson's Disease: A Network Meta-Analysis
Source: Front Aging Neurosci. 2019 Feb 22;11:23. doi: 10.3389/fnagi.2019.00023 (PMC6395396; doi:10.3389/fnagi.2019.00023)

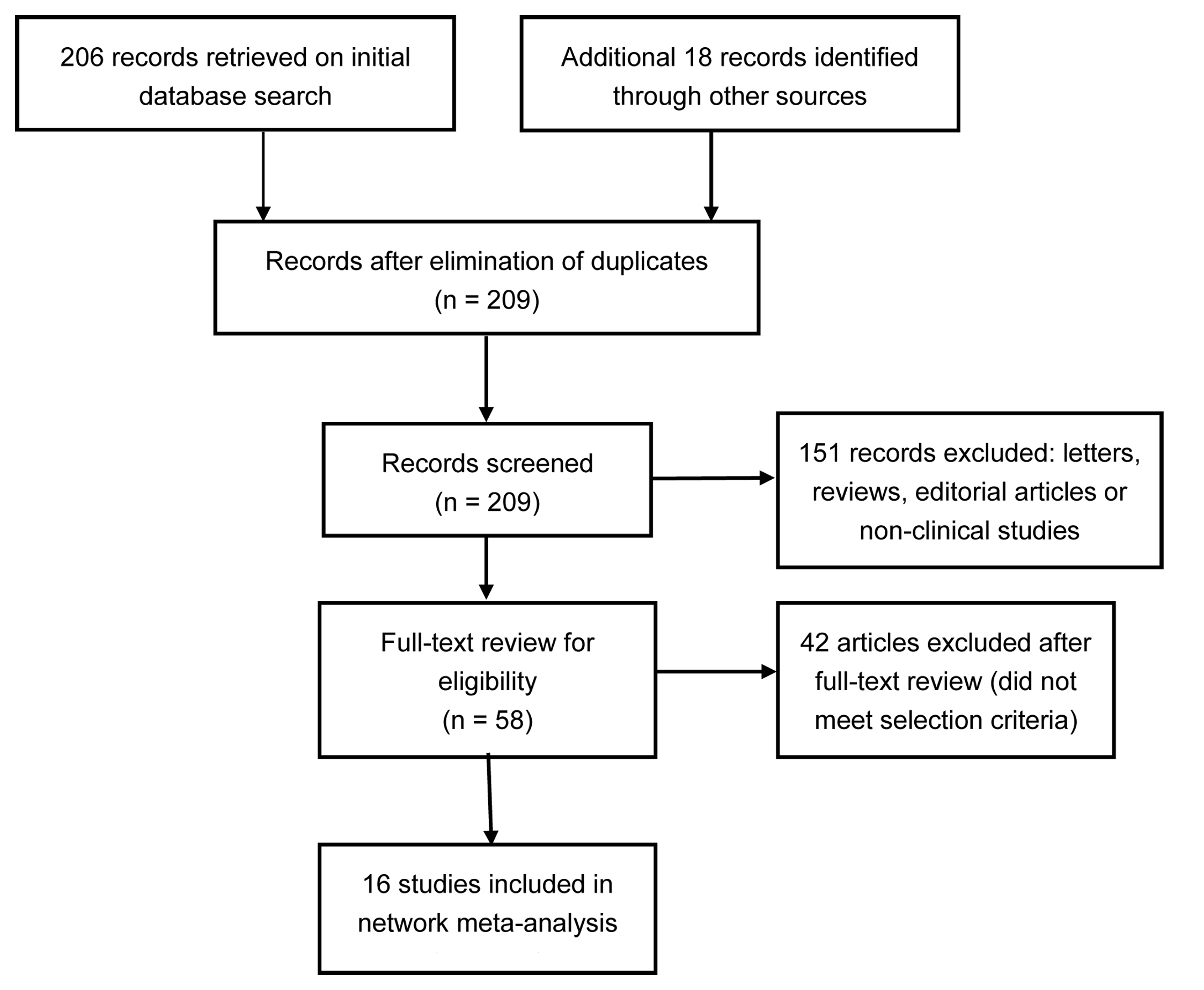

Supplement: Figure S1 — Schematic illustration of the literature search and study selection criteria. [file Image_1.TIF]

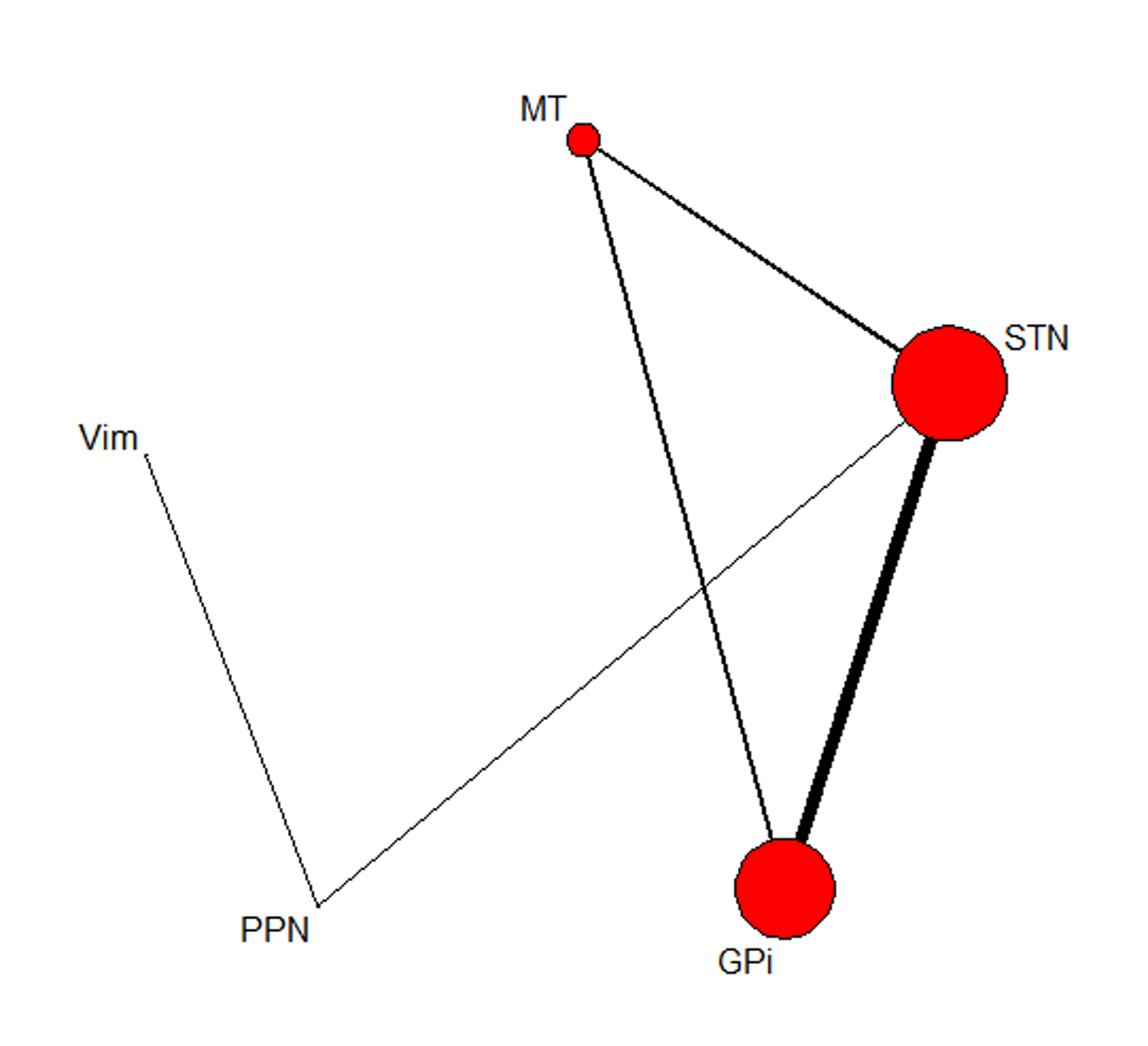

Supplement: Figure S2 — Evidence network for different DBS (Deep brain stimulation) targets. STN, subthalamic nucleus; GPi, internal globus pallidus; Vim, ventral intermediate nucleus; PPN, pedunculopontine nucleus; MT, medical therapy. [file Image_2.TIF]

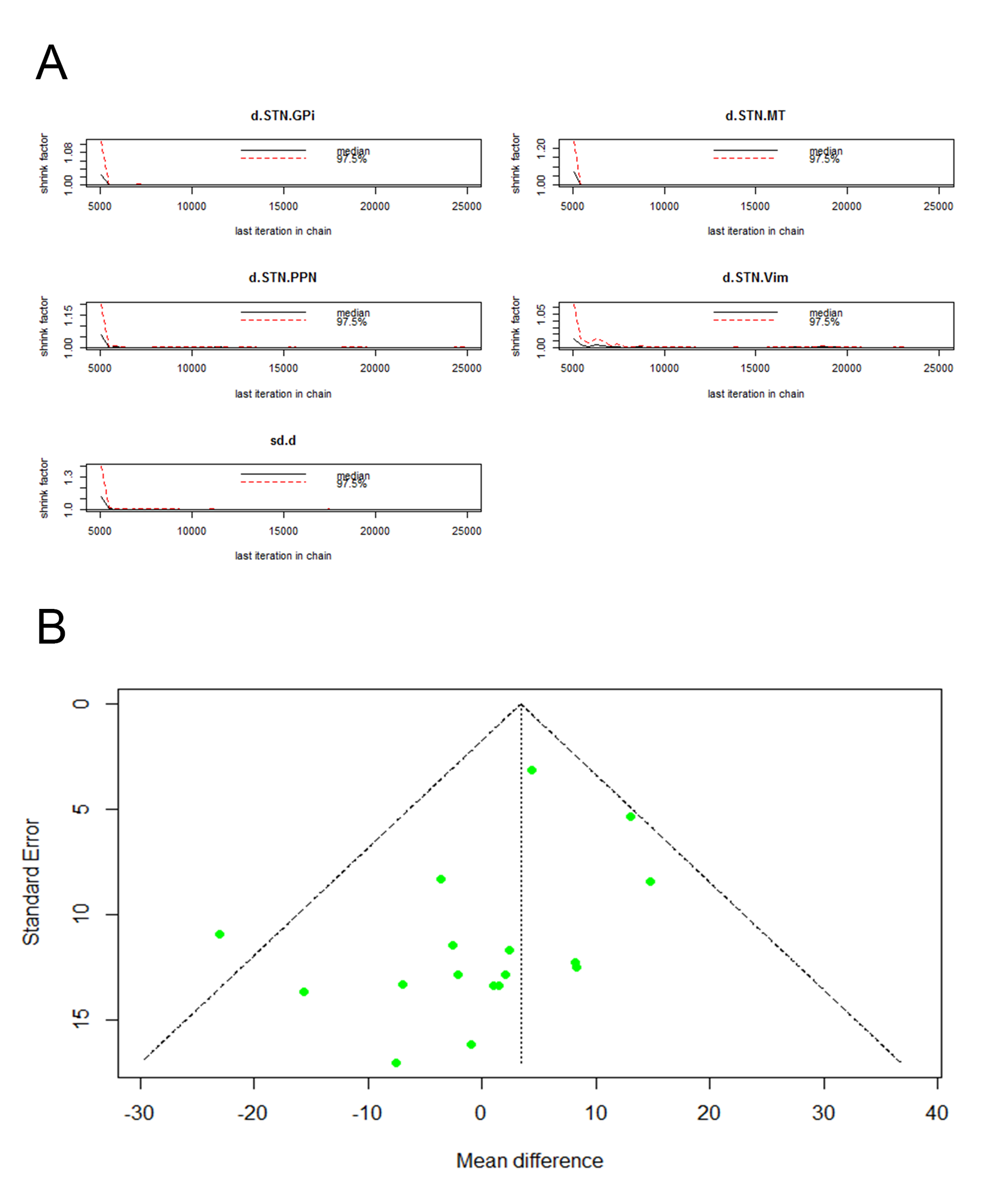

Supplement: Figure S3 — STN-subthalamic nucleus, GPi-internal globus pallidus, Vim-ventral intermediate nucleus, PPN-pedunculopontine nucleus, MT-medical therapy, sd-standardized mean. [file Image_3.TIF]
